# Books and Magazines

**Published:** 1906-08

**Authors:** 


### Books and Magazines.

CARR'S PEDIATRICS—THE PRACTICE OF PEDIATRICS BY EMINENT AUTHORITIES. Edited by Walter Lester Carr, M. D., Consulting Physician to the French Hospital; Visiting Physician to the Infants' and Children's Hospital, New York. In one very handsome octavo volume of 1014 pages with 199 engravings and 32 full-page plates in colors and monochrome. Cloth, $6, net; leather, $7, net; half Morocco, $8, net. Lea Brothers & Co., Publishers, Philadelphia and New York, 1906.

This is one of a series of three volumes dealing respectively with Gynecology, Obstetrics, and Pediatrics, and jointly, covering this whole domain in the light of the world's latest and best knowledge. The volume on Gynecology was published in May.

The volume on Pediatrics, now in the reader's hands, is from the pens of well-known authorities in America and England, who have been selected as peculiarly well fitted to write on the subjects as-

### The New York Polyclinic Medical School and Hospital.

[UNIVERSITY OF THE STATE OF NEW YORK.]

signed to them. The authors have kept in mind first, the clinical picture of each disease, and second, the best methods for its treatment. This plan has allowed each contributor to give his own observations and the therapeutic measures which have produced the most satisfactory results. Naturally this adds to each contribution a personal element which is entitled to consideration, as the authors are, without exception, clinicians and teachers of wide experience.

In the arrangement of the volume more space than usual has been allotted to infant feeding, diseases of the alimentary tract, disorders of nutrition, respiration, and circulation, and to contagious diseases, the object being to describe the conditions most intimately associated with disease in children and not those which are more common in adult life and found but rarely in childhood. In some sections extra space has been given to methods of diagnosis which are now regarded as essential by physicians who wish to be exact in their work, but the details of which are not readily accessible elsewhere. On the other hand, mooted pathological questions have been omitted, and the pathology stated by each author is limited to what is regarded as essential for a comprehensive knowledge of the disease with which it is associated.

Under the able editorship of Dr. Carr this great fund of information has been arranged most conveniently for reference and study; over-lapping and repetition, so frequently found in composite books, has been avoided, and the volume furnishes a veritable encyclopedia of our present-day knowledge in Diseases of Children, charmingly written and rich with the finest examples of the illustrator's art.

It is manifestly to the advantage of every physician to have this entire series of three volumes at hand, but the publishers, having in mind the convenience of those who are interested in one or two individual departments, have issued each volume as a separate book, complete in itself, and either volume of the series may be purchased separately.

The completing volume of this series (Peterson's Obstetrics) will be published in August.

---

A Treatise on Surgery. In two volumes. By George R. Fowler, M. D., Examiner in Surgery, Board of Medical Examiners of the Regents of the University of the State of New York; Emeritus Professor of Surgery in the New York Polyclinic, etc. Two imperial octavos of 725 pages each, with 888 text illustrations and 4 colored plates, all original. Philadelphia and London: W. B. Saunders Co., 1906. Per set: Cloth, $15, net; half Morocco, $17, net.

We have been looking forward to the appearance of this work with the greatest expectations, for Dr. Fowler's endeavors in the field of practical surgery have been such as to stamp his writings with unquestionable authority. It is not too much, indeed, we feel it is too little, to say that our expectations have been fully realized. The work is a masterpiece. It is an accurate, up-to-date treatise on surgery, skillfully presented. This entirely new work presents the science and art of surgery as it is practiced today. The first part of the work deals with general surgery, and embraces what is usually included under the head of principles of surgery. Special attention is given to the subject of inflammation from the surgeon's point of view, due consideration being accorded the influences of traumatism and bacterial infection as the predisposing and exciting causes of this condition. Then follow sections on the injuries and diseases of separate tissues, gunshot injuries, acute wound diseases, chronic surgical infections (including syphilis), tumors, surgical operations in general, foreign bodies, and bandaging. The second part of the work is really the clinical portion, devoted to regional surgery. Herein the author especially endeavors to emphasize those injuries and surgical diseases that are of the greatest importance, not only because of their frequency, but also because of the difficulty of diagnosis and the special care demanded in their treatment. Throughout special attention has been given to diagnosis, the section on laboratory aids being unusually excellent. The text is elaborately illustrated with entirely new and original illustrations, and evidently neither labor nor expense has been spared to bring this feature of the work up to the highest standard of artistic and practical excellence.

---

THE HEALTH-CARE OF THE BABY—A HANDBOOK FOR MOTHERS AND NURSES. By Louis Fischer, M. D. 12mo, cloth, 166 pages, 75 cents, net; by mail, 82 cents. Funk & Wagnalls Co., New York.

Dr. Fischer is a specialist on children's diseases, being attending physician to the Willard Parker and Riverside Hospitals; former Instructor in Diseases of Children at the New York Post-Graduate Medical School and Hospital. He is also the author of "Infant Feeding in Health and Disease," "A Text-Book on Diseases of Infancy and Childhood," etc. The present work, which is a handbook for mothers and nurses, will be especially timely now that the baby's most trying period—hot weather—is at hand. The book covers the subjects of feeding in health and disease; gives directions for the management of fever, and is a guide during such diseases as measles, croup, skin diseases, etc. It gives ample advice in cases of accidents, poisoning, etc. The correction of bad habits, and the management of rashes, have received careful consideration.

Consumption: Its Relation to Man and His Civilization—Its Prevention and Cure. By John Bessner Huber, A. M., M. D., Fellow of New York Academy of Medicine; Member of the National Association for the Study and Prevention of Tuberculosis; Visiting Physician to St. Joseph's Hospital for Consumptives; Member of the Advisory Board, the New Mexico Sanatorium, etc. Cloth, 525 pages. Price not stated. J. B. Lippincott Co., Philadelphia and London, 1906. Just issued.

This book is most timely, as never before in the history of the world has there been such widespread interest in the study of this dread disease in the endeavor to devise and put into execution measures for its restriction and prevention. And it seems to be very thorough in its scope and aim, teeming with valuable data gleaned from a wide field of observation. The author says: "It is a comprehensive exposition of the effects which consumption has had upon civilization and a consideration of its relation to human affairs." * * * "Manifestly, medical science can not cope alone and unaided with this difficult and prodigious world-problem; many forces, economic, legislative, sociological, humanitarian, must be enlisted."

It is interesting and important to note that the fourth biennial session of the American International Congress on Tuberculosis will be held in New York City in November, 1906, for the purpose of dealing with the subject in the relations above mentioned. It is a medico-legal subject, and the Congress will endeavor mainly to arouse the people and the governments, National and State, to action, to limit the spread and so far as authority, money and united efforts can do so, remove the principal causes of the disease.

---

THE PRACTICAL MEDICINE SERIES.—Comprising ten volumes on the year's progress in Medicine and Surgery. Under the general editorial charge of Gustavus P. Head, M. D., Professor of Laryngology and Rhinology, Chicago Post-Graduate Medical School. Vol. 1: General Medicine; edited by Frank Billings, M. S., M. D., Head of the Medical Department and Dean of the Faculty of Rush Medical College, Chicago, and J. H. Salsbury, A. M., M. D., Professor of Medicine, Chicago Clinical School; Series 1906. Vol. 2: General Surgery: Edited by Jno. B. Murphy, A. M., M. D., LL. D., Professor of Surgery in Rush Medical College (in affiliation with University of Chicago). Vol. 3: The Eye: Casey A. Wood, C. M., M. D., D. C. L., Professor of Clinical Ophthalmology, Medical Department University of Illinois, etc., etc. The Ear: Albert H. Andrews, M. D., Professor of Otology, Chicago Post-Graduate Medical School, etc. etc. The Nose and Throat: G. P. Head, M. D. (*vide supra*). Year Book Publishing Co., 40 Dearborn St., Chicago. Price, per volume, $1.25; the set of ten volumes, $10.

These three volumes are of a series of ten issued at about monthly intervals, and covering the entire field of medicine and surgery. Each volume being complete for the year prior to its publication on the subject of which it treats. The series is published primarily for the general practitioner, at the same time the arrangement in several volumes enables those interested in special subjects to buy only the parts they desire.

